# Crossing the Blood-Brain Barrier: Advances in Nanoparticle Technology for Drug Delivery in Neuro-Oncology

**DOI:** 10.3390/ijms23084153

**Published:** 2022-04-09

**Authors:** Andrew M. Hersh, Safwan Alomari, Betty M. Tyler

**Affiliations:** Department of Neurosurgery, Johns Hopkins University School of Medicine, Baltimore, MD 21287, USA; ahersh2@jh.edu (A.M.H.); salomar1@jhmi.edu (S.A.)

**Keywords:** blood-brain barrier, nanoparticle, drug delivery, tumor

## Abstract

The blood-brain barrier (BBB) constitutes a microvascular network responsible for excluding most drugs from the brain. Treatment of brain tumors is limited by the impermeability of the BBB and, consequently, survival outcomes for malignant brain tumors remain poor. Nanoparticles (NPs) represent a potential solution to improve drug transport to brain tumors, given their small size and capacity to target tumor cells. Here, we review the unique physical and chemical properties of NPs that aid in BBB transport and discuss mechanisms of NP transport across the BBB, including paracellular transport, carrier-mediated transport, and adsorptive- and receptor-mediated transcytosis. The major types of NPs investigated for treatment of brain tumors are detailed, including polymeric NPs, liposomes, solid lipid NPs, dendrimers, metals, quantum dots, and nanogels. In addition to their role in drug delivery, NPs can be used as imaging contrast agents and can be conjugated with imaging probes to assist in visualizing tumors, demarcating lesion boundaries and margins, and monitoring drug delivery and treatment response. Multifunctional NPs can be designed that are capable of targeting tumors for both imaging and therapeutic purposes. Finally, limitations of NPs for brain tumor treatment are discussed.

## 1. Introduction

Delivery of therapeutics to the central nervous system (CNS) is limited by the blood-brain barrier (BBB), a microvascular network that separates the CNS from the peripheral blood circulation [1]. The BBB tightly regulates transport of molecules into the brain to maintain homeostasis and shield the CNS from toxins, allowing an optimal environment for neuronal signaling [2]. Endothelial cells lining the BBB lack fenestrations and are connected via specialized tight junctions (TJs) that include occludins, claudins, and junction adhesion molecules spanning the membrane. ZO-1, ZO-2, ZO-3, and cingulin act as cytosolic linkers connecting transmembrane TJ proteins to actin [3,4,5]. Adherens junctions, consisting of cadherins connected to actin via alpha, beta, and gamma catenins, are also present at the BBB [4]. The TJs and adherens junctions restrict paracellular transport of ions, polar solutes, and most macromolecules [3,4,5]. Ion channels and transporters carefully regulate the concentration of ions to promote optimal synaptic transmission, while transport proteins promote uptake of specific solutes and nutrients, such as glucose and amino acids [5]. The endothelial cells interact with the basement membrane and form a neurovascular unit along with astrocytes, pericytes, and the extracellular matrix, which provides structural and functional support [6,7]. Uptake of most macromolecules can only occur by transcytosis [5,8]. The BBB also includes multidrug efflux transporters which actively remove undesired molecules from the brain [8,9].

Malignant brain tumors carry a poor prognosis, and therapeutic treatment is limited by BBB impermeability. Glioblastoma is the most common brain malignancy and represents the most aggressive form of glioma [10]. Surgical resection is typically the standard of care for patients with malignant brain tumors, and more extensive resections have been shown to improve survival outcomes [11]. However, achieving complete resection is not always feasible for tumors located in regions whose resection poses risk of substantial postoperative neurological deficits [11]. Radiotherapy with adjuvant temozolomide, an anticancer drug, generally follows surgical resection and has also been shown to improve outcomes [12]. In addition, wafers can be implanted at the surgical site to release chemotherapeutic drugs, such as carmustine [13]. Despite the combination of surgery, radiation, and chemotherapy, overall outcomes remain poor for patients, with a 5 year survival rate of nearly 32% for malignant brain tumors, which decreases to 5% for glioblastoma [14,15]. The BBB remains impermeable to nearly all large macromolecules and excludes nearly 98% of small-molecule drugs from the brain, limiting available therapeutic regimens [16]. Furthermore, radiation and chemotherapy are associated with substantial systemic side effects [17]. Notably, brain neoplasms, particularly glioblastoma, can be associated with decreased expression of claudins and occludins at TJs, while an increase in vascular endothelial growth factor promotes heterogeneous and leaky neovascularization. These vascular changes constitute the blood-brain tumor barrier (BBTB) and are associated with increased permeability relative to the BBB; nonetheless, the increase in permeability is countered by upregulation of solute carriers and efflux transporters that remove therapeutic agents from the tumor. Additionally, the breakdown in TJs primarily occurs at the tumor core, and peripheral margins remain resistant to uptake of macromolecules [18]. 

Therefore, novel strategies are needed for therapeutic targeting of drugs to brain tumors. Non-invasive mechanisms include chemical disruption of the BBB using vasoactive compounds to induce an inflammatory reaction or hyperosmolar compounds to damage endothelial cells [18], focused ultrasound-mediated reversible disruption using thermal ablation or acoustic cavitation [18,19,20], intranasal delivery [21], suppression of efflux pump inhibitors [22], viral vectors such as adeno-associated viruses [23], and molecular “Trojan horse” proteins that bind BBB receptors and facilitate delivery across transporters [24]. Despite promising research in animal models, few methods have achieved clinical success.

Nanoparticles (NPs) represent a novel approach for crossing the BBB and include a diverse array of compounds whose unique physical and chemical properties enable targeted therapeutic delivery to brain tumors. NPs are particularly advantageous due to their small size, low level of toxicity, and controlled drug release profile [25]. Additionally, their surface can be readily modified with proteins that target specific receptors to localize drug delivery [26]. Some malignant brain tumors are associated with disruptions in the BBB which allow for enhanced NP transport compared to larger molecules [27]. NPs exploit both active and passive transport mechanisms, including passive diffusion, carrier-mediated transport, and transcytosis [28]. Various NPs are available for drug delivery, including polymeric NPs, liposomes, dendrimers, inorganic particles, quantum dots, and thermosensitive pastes. Here, we review the different types of NPs studied for drug delivery across the BBB and discuss their physical principles and transport strategies.

## 2. Properties of Nanoparticles

### 2.1. Size and Charge of NPs

NPs are small molecules ranging in size from 1 to 1000 nm. Their small size is advantageous for crossing the BBB, and studies have shown increasing permeability through BBB gaps as NP size decreases, with essentially no permeability above 200 nm [29,30]. However, renal filtration rapidly clears NPs < 5 nm [31]. Consequently, most studies targeting drug delivery across the BBB use NPs with diameters between 10–100 nm [32]. For example, Ohta et al. illustrated that 15 nm gold NPs had higher delivery efficiency into the mouse brain compared to 3 nm and 120 nm particles [33]. Moreover, NPs must diffuse through the brain extracellular space after transport across the BBB. This space between cells constitutes approximately 20% of total brain volume and is commonly estimated at 20 nm in width, although its true dimensions in vivo are difficult to compute. Consequently, the diffusion of larger NPs will be limited by the size of the extracellular space [34]. The optimal size is dependent on the type of NP, associated surface proteins and coating, and physiological functioning of the BBB, and therefore should be determined for each NP-based therapeutic platform. 

Electrostatic interactions between NPs and the BBB also influence permeability. Negatively charged proteoglycans confer a high density of anionic surface charges to the endothelial cells of the BBB. Therefore, positively charged particles have favorable electrostatic interactions with endothelial cell membranes and are best suited for adsorptive-mediated transcytosis across the BBB [35]. In contrast, neutral particles are less permeable than positively charged NPs by about 100-fold [36]. However, a study of the rat brain by Lockman et al. indicated that cationic NPs can exert a toxic effect on the BBB and disrupt its integrity, whereas neutral NPs and low concentrations of anionic NPs had no such effect on BBB integrity [37]. Similarly, Knudsen et al. illustrated that direct intracerebroventricular injection of cationic NPs into the rat brain results in a greater loss of neurons compared to anionic NPs; however, the effects of intravenous administration, which requires traversing the BBB, are unclear [38]. Positively charged NPs may result in the formation of reactive oxygen species, which can damage cells and lead to necrosis or apoptosis [39,40]. Given the resistance of the BBB’s endothelial cells to anionic charges, cationic NPs may represent a potential delivery mechanism for negatively charged genetic material such as small-interfering RNA for targeted gene therapy to tumors [41,42]. 

### 2.2. Ligands and Functional Groups

The surfaces of NPs can be conjugated with specific ligands, including peptides, proteins, antibodies, and surfactants, to enhance BBB crossing by improving circulation time or by binding to endothelial receptors [43]. Increasing the ligand density improves polyvalency and avidity, resulting in a higher probability of internalization by endothelial cells [44]. However, an excessively high density can result in steric hindrance, decrease the diffusion coefficient, and increase NP size. Additionally, intracellular trafficking modalities, such as clathrin-mediated endocytosis and pinocytosis, can vary depending on ligand density [45]. Finally, an excessive ligand density may prevent NP release from cell surfaces and impair exocytosis due to the high avidity [46]. Therefore, an optimal ligand density should be considered in NP formulation, one with sufficient affinity and avidity to promote internalization but not too high that internalization is impaired. For example, Anraku et al. demonstrated that a NP with glucose molecules that recognize the glucose transporter-1 receptor on endothelial cells showed optimal BBB permeability at 25% surface glucose compared to either 10% or 50% surface density [47].

Chains of polyethylene glycol (PEG), an inert polymer, are commonly added to NPs to increase circulation time by preventing opsonization, phagocytosis, and capture of NPs by the reticuloendothelial system (RES). PEG is considered a “stealth” molecule and provides a longer window of opportunity for the NPs to interact with endothelial receptors [48,49]. Nance et al. also illustrated that a dense coating of PEG can allow for penetration of NPs as large as 114 nm which otherwise would not cross the BBB [50]. Furthermore, it has been illustrated that the addition of PEG chains to lipid NPs can prevent microglia-mediated inflammatory responses otherwise seen with unmodified lipid NPs [51]. However, excessively long chains hinder BBB penetration by increasing PEG flexibility such that the folded chains reduce ligand exposure and produce steric hindrance [52]. An optimal length should be considered with NP PEGylation, one that is neither too short nor too long.

Other molecules, including peptides and proteins, can be added to NP surfaces to improve transcytosis by targeting specific cellular receptors on the BBB, as well as targeting specific tissue [53]. These ligands are often termed “Trojan horses” as they are recognized and internalized by BBB receptor-mediated transport systems along with their associated NP [54,55,56]. A classic well-studied example is the use of transferrin (Tf), which is used by the body to transport iron across the BBB. The transferrin receptor (TfR) is found in abundance on endothelial cells of the BBB and internalizes Tf via receptor-mediated endocytosis [53,57,58]. Endogenous Tf competes with Tf bound to NPs for saturation of the TfR, but antibodies with high binding affinity or those that bind other epitopes can be used, such as the OX26 anti-TfR monoclonal antibody, which improves NP permeability across the rodent BBB compared to Tf-conjugated NPs [59,60,61]. Other antibodies and proteins include those targeting the leptin receptor [62,63], low-density lipoprotein receptor (LDL) [64,65], and the insulin receptor [66]. Proteins can be selected to target specific brain regions. For example, Betzer et al. found that insulin-coated NPs accumulated in high levels in the hippocampus, where insulin receptors are abundant [67]. Additionally, ligands that respond to environmental stimuli can improve NP targeting, such as pH-sensitive moieties that can be cleaved within the acidic microenvironment of tumor cells to promote drug release [68]. 

Small molecules can also be conjugated to NPs to enhance binding affinity and target receptors. The nucleoside adenosine is a neuromodulator involved in neuronal and synaptic function and consequently is a pharmacological agent of interest for neurological diseases [69,70]. However, its short circulation time renders it ineffective at crossing the BBB [71]. Gaudin et al. showed that conjugation of adenosine to squalene NPs protected adenosine from metabolization and increased circulation time, resulting in improved neurological outcomes in a rodent model of spinal cord injury [72]. Adenosine receptor ligands have also shown efficacy in increasing BBB permeability by reducing tight junction cohesion [73]. Glucose molecules can be added to NPs to facilitate transport across glucose-transporters, which are often upregulated in tumor cells [74]. Other small molecules investigated for NP delivery include glutathione and maltobionic acid [41]. Aptamers, or short single-stranded nucleotide sequences capable of unique structural conformations, can also be used given their thermostability, modifiability, and high specificity and binding affinity for proteins and cells [75,76,77,78]. 

## 3. Nanoparticle Transport Mechanisms

**Figure 1:**
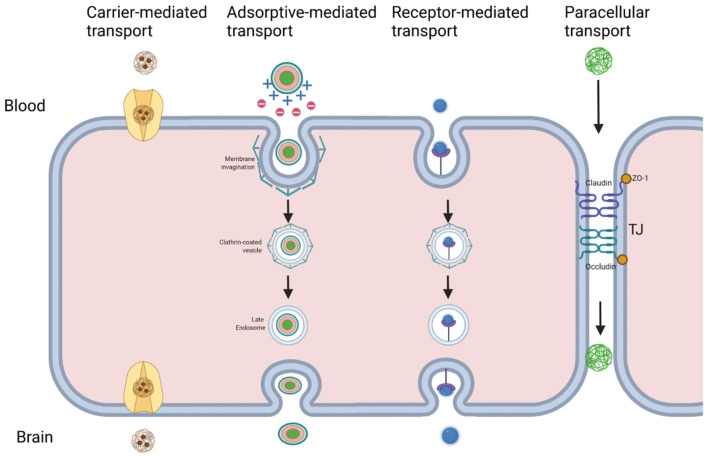
NPs can exploit several transport mechanisms to cross the BBB. Their small size is advantageous for paracellular transport across the TJs. Carrier-mediated transport takes advantage of endogenous BBB transporters needed for entry of molecules for homeostasis and neuronal health. Adsorptive-mediated transport processes occur via favorable interactions between the surfaces of NPs and the endothelial membrane, while receptor-mediated processes stem from recognition of a ligand on the NP by a membrane receptor. Membrane invagination results in internalization of the NPs into clathrin-coated pits or caveolae before exiting into the target tissue.

NPs are generally administered intravascularly. Intraventricular administration can be employed to improve drug delivery and bypass the BBB; however, this method is more invasive than intravascular delivery and is limited by rapid turnover of cerebrospinal fluid [26,79,80,81]. Direct intraparenchymal injection has been explored for drugs, but requires patients to undergo surgical operations [82]. Intranasal delivery of NPs via the olfactory nerve pathway has also been explored, although clearance is reduced by the ciliary movement [83,84]. Once NPs arrive at the BBB, they can cross using several strategies, including passive diffusion, carrier transport, and adsorptive- and receptor-mediated transcytosis (Figure 1). Techniques that disrupt BBB integrity also facilitate NP transport.

Small lipophilic cationic NPs can passively diffuse across the cell membranes of BBB endothelial cells [85]. This is a spontaneous but uncommon process at the BBB; however, NPs can exploit this mechanism by virtue of their small size. Cationic charges and lipid molecules can be added to NPs to increase their diffusion capacity [86]. Gold NPs in particular have been shown to cross the BBB using passive diffusion across endothelial cells [87]. NPs can also be targeted to specific carrier transporters by conjugating an appropriate ligand, such as glucose for targeting the glucose transporter [88]. 

### 3.1. Adsorptive- and Receptor-Mediated Transcytosis

Active transport of NPs from the apical to basolateral plasma membranes of endothelial cells is a common transport mechanism at the BBB, occurring via adsorptive- or receptor-mediated transcytosis [8]. Adsorptive-mediated transcytosis (AMT) stems from interactions between the surface properties of NPs and the endothelial plasma membrane [89]. These interactions result in invagination of the cell membrane and vesicle formation mainly through negatively charged clathrin-coated pits, as well as through caveolae. Endosomes are generated for direct trafficking of NPs either to the basolateral surface, lysosomes for destruction, or back to the apical plasma membrane [8,90]. Electrostatic interactions between cationic NPs and the anionic plasma membrane are commonly implicated in AMT [91]. Adding cationic charges to NPs and conjugating compounds such as lectin, cardiolipin, and heparin have been shown to induce BBB adsorption when conjugated to NPs [92]. As a non-specific process, AMT is limited as a strategy for targeting specific brain tissue, as cationic NPs can be adsorbed by numerous anionic cell membranes [92]. 

In contrast, receptor-mediated transcytosis (RMT) involves the binding of NPs to specific receptors at the apical surface of endothelial cells via unique ligands that promote endocytosis. Intracellular transport vesicles are formed by invagination of the cell membrane, similarly to AMT, and the NP is transported to the basolateral surface [93]. RMT is employed for the homeostatic shuttling of nutrients such as iron, insulin, and leptin, and NPs can be conjugated either to natural or artificial ligands. These ligands function as “Trojan horses” to allow NP access to the brain. RMT allows for specific targeting of brain tissue and tumor cells [94]. NPs can be localized to specific brain tissues by designing ligands that can attach both to receptors on the apical surface of BBB endothelial cells and target tissue, including the transferrin and low-density lipoprotein receptors, which are found at the BBB and are over-expressed in cancer cells [95,96,97]. For example, Liang et al. illustrated that conjugation of the chemotherapeutic vincristine sulfate to LDL particles could specifically bind the LDL receptor and target glioma cells in mice [98]. However, NPs with excessive avidity may remain bound to the endothelial cells rather than detaching from their receptor, an important consideration when designing NPs and conjugating ligands [99].

### 3.2. BBB Disruption to Improve NP Permeability

In addition to passive and active transport mechanisms, NP permeability can be increased by strategies that disrupt BBB integrity. Infusion of osmotic agents, such as intracarotid injection of hyperosmolar mannitol, can cause endothelial dehydration and reversibly disrupt BBB integrity to improve delivery of chemotherapeutic agents to the brain [100,101]. Boockvar et al. conducted a clinical trial on 30 human patients with recurrent malignant glioma, illustrating that infusion of the chemotherapeutic bevacizumab after osmotic mannitol disruption can decrease tumor enhancement and perfusion [102]. Molecular substances can also modulate BBB permeability, such as leukotrienes, bradykinin, and adenosine, likely by inducing a temporary inflammatory reaction and improving paracellular permeability [18,103,104]. Laser-induced thermal therapy and laser stimulation of NPs have also been explored to improve paracellular diffusion of NPs by increasing permeability through tight junctions [105,106]. However, care must be taken to evaluate the safety of approaches that disrupt the BBB, which can allow an influx of other compounds that contribute to cerebral toxicity [107].

Focused ultrasound (FUS) has also illustrated promise as a tool to disrupt BBB integrity and improve NP penetration. The procedure involves systematic administration of microbubbles followed by pulses of FUS, producing oscillations in the microbubbles. These oscillations produce a physical cavitation effect that results in the stretching of vessel walls and detachment of tight junctions [108]. FUS non-invasively downregulates occludins and claudins at the tight junctions of the BBB while increasing vesicular transport vehicles such as caveolins, thereby improving both paracellular transport and transcytosis of therapeutics [109,110]. FUS is advantageous in targeting specific regions of brain tissue, whereas osmotic or chemical modification can affect BBB permeability over a widespread area [111]. Disruption of the BBB can last several hours after sonication [112]. The safety and efficacy of FUS for BBB disruption has been established but depends on several parameters, including burst length and pulse frequency [113]. FUS transducers can be implanted in the skull to improve delivery of chemotherapeutics [114,115]. FUS can also be guided using Magnetic Resonance Image (MRI) technology to improve targeting of specific tissue and has been shown to improve BBB permeability to NPs, enhance molecular imaging of tumors, and reduce tumor invasiveness and growth, although this work has mainly been performed in rodent models [116,117,118,119]. For example, Treat et al. illustrated that FUS treatment of NPs with the chemotherapeutic drug doxorubicin reduces gliosarcoma growth compared to doxorubicin alone and improves median survival in a rat glioma model [120]. A Phase I study in five patients showed that MR-guided FUS can temporarily open the BBB and improve delivery of chemotherapeutics [121].

Unique structural changes along the BBTB promote an increase in permeability of molecules. ATP-sensitive potassium channels and calcium-dependent potassium channels are upregulated along the BBTB and can be targeted by agonists to further increase permeability [122,123,124,125]. Activation of calcium-dependent potassium channels can increase the formation of endothelial pinocytic transport vesicles to improve drug transport across the BBTB [123]. Additionally, there is a loss of TJs and endothelial cell adhesions in cancer, along with a neuroinflammatory milieu and increase in cytokine receptors which increase BBTB permeability [126]. The loss of TJs allows for greater paracellular diffusion of small compounds, although a concomitant upregulation of efflux pumps removes many larger therapeutic agents from the tumor [18]. Additionally, the heterogeneous nature of the BBTB results in uneven distribution of therapeutic agents to the tumor [105]. However, NPs loaded with chemotherapeutic drugs have shown enhanced efficacy in targeting tumor tissue compared to healthy tissue due to the increased permeability and retention of drugs in tumor tissue, a finding known as the enhanced permeability and retention effect [127,128]. Consequently, NPs loaded with anti-cancer agents have been repeatedly shown to exert stronger anti-glioma effects compared to the free agent alone, making NPs important agents for targeting brain tumors [129].

## 4. Types of NPs

NPs can adopt a wide range of configurations, each with unique chemical and physical properties for improving access to brain tumors (Table 1). Here, we review the main categories of NPs investigated for treatment of brain neoplasms. 

### 4.1. Polymeric NPs

Polymeric NPs consist of a core-shell structure with hydrophilic polymers coating the surface to provide stability and reduce phagocytosis while the interior core includes a polymeric matrix. The drug can be encapsulated in the core or conjugated to the surface [162]. Following uptake by the target tissue, the drug is released from the surface, or the polymeric matrix is triggered to release the drug [41]. Important features of polymeric NPs include stability, non-toxicity, biodegradability, biocompatibility, and simple manufacturing processes, offering advantages over non-polymeric NPs [162,163]. Two common polymeric NPs include poly(butyl cyanoacrylate) (PBCA) and poly(lactic acid) (PLA), with PBCA offering fast biodegradability [163]. Poly(lactic-co-glycolic acid) NPs can also be synthesized, consisting of PLA and poly(glycolic acid), with the copolymer ratio determining the degree of hydrophobicity and consequently degradation rate [41,164,165]. Polysaccharides such as chitosan and hyaluronic acid and proteins such as albumin are also commonly featured in polymeric NPs [166]. Polymeric NPs can be readily modified with ligands such as Tf or PEG which can improve bioavailability and targeting of the NPs to specific brain tissue, while surfactants such as polysorbate 80 can improve RMT by the LDL receptor due to apolipoprotein adsorption onto the NPs [41].

The use of polymeric NPs to improve chemotherapeutic access to brain tumors has been extensively explored. PLA nanoparticles coated with Tf and loaded with the anti-cancer agent 3-bis(2-chloroethyl)-1-nitrosourea have been shown to significantly improve survival in a rat glioma model [130,131]. Additionally, doxorubicin bound to PBCA accumulates in the rat brain after intravenous administration at greater concentrations than seen with administration of doxorubicin alone, and results in less cardiotoxicity and cytotoxicity [132,133]. Conjugation of Tf to a PEG-PLA NP carrying doxorubicin can improve the anti-cancer effects of doxorubicin in a rat model compared to the unconjugated NP or doxorubicin alone [167]. PBCA NPs can also be coated with the surfactant polysorbate 80 and deliver intravenous methotrexate across the BBB, with increased drug levels seen for smaller-sized NPs [134]. Similarly, PBCA NPs coated with polysorbate-80 carrying the drug gemcitabine improved survival time in a rat in vivo brain tumor model [136]. They can also be used to transport the chemotherapeutic agent temozolomide across the BBB [135]. Other types of polymeric NPs have also been tested, including a NP based on serum albumin, which was shown to be capable of carrying siRNA targeting STAT3, a transcription factor involved in glioblastoma progression, thereby improving survival in mouse models [168].

### 4.2. Liposomes and Solid Lipid NPs

NPs can be designed as closed spherical vesicles known as liposomes with one or more lipid bilayers composed of natural or synthetic phospholipids enclosing a discrete aqueous space. Liposomes can transport both hydrophobic and hydrophilic therapeutics—the former in the lipid bilayer, and the latter in the aqueous phase [169]. Together with polymeric NPs, liposomes have been extensively explored for crossing the BBB [170]. The amphiphilic phospholipids spontaneously associate into bilayers, and their composition governs the overall properties of the liposome, including permeability and steric hindrance [171]. They are readily modifiable and are biocompatible with low toxicity, and can be conjugated with ligands, such as PEG for steric stabilization, to reduce clearance by phagocytosis [169,171,172,173]. Liposomes can also encapsulate imaging agents to track drug delivery or visualize tumors [174]. Solid lipid NPs (SLNs) are similar to liposomes but consist as spheres of solid lipids with a strongly lipophilic matrix, instead of a lipid bilayer [171]. Compared to liposomes, they are easier to produce, exhibit greater efficiency at drug transport, and are more stable [175]. They can be derived from fatty acids, fatty alcohols, glyercides, and waxes [175].

Liposomes have been widely studied for treatment of gliomas in rat and mouse models, and several human clinical trials have been performed [176]. Liposomal doxorubicin is an approved medication used to treat ovarian cancer, multiple myeloma, Kaposi sarcoma, and other neoplasms [177]. The drug has also been explored for glioblastoma and metastatic brain tumors, with human trials showing selective accumulation in tumors [178,179]. Improved delivery of the chemotherapeutic irinotecan across the BBB can improve survival time from 29.5 days with free irinotecan to 54.2 days with the liposome in a rat brain tumor model [140]. FUS has been used to improve BBB permeability to liposomes carrying drugs, including cisplatin or doxorubicin, resulting in reduced tumor growth in mice [120,180], while radiotherapy combined with liposomes carrying carboplatin improved survival time in rats [181]. Similarly, liposomes carrying paclitaxel were shown to cross the BBB and target glioma tumors in rats [182]. Modifications can be readily made to improve their clinical effect. For example, conjugating integrin αvβ3-specific vector to liposomes carrying paclitaxel was shown to target gliomas and improve survival in mice by targeting integrin receptors overexpressed on glioma cells and present on endothelial BBB cells [183]. Conjugation of tamoxifen to liposomes carrying topotecan to brain tumors can improve survival time in rat models by inhibiting endothelial drug efflux transporter at the BBB [142]. Cationic lipids can also carry DNA and RNA for use in gene therapy or RNA interference [176,184,185]. Liposomes can also be encapsulated with radionuclides, such as rhenium-186, to target gliomas with radiation therapy [186,187].

### 4.3. Dendrimers

**Figure 2:**
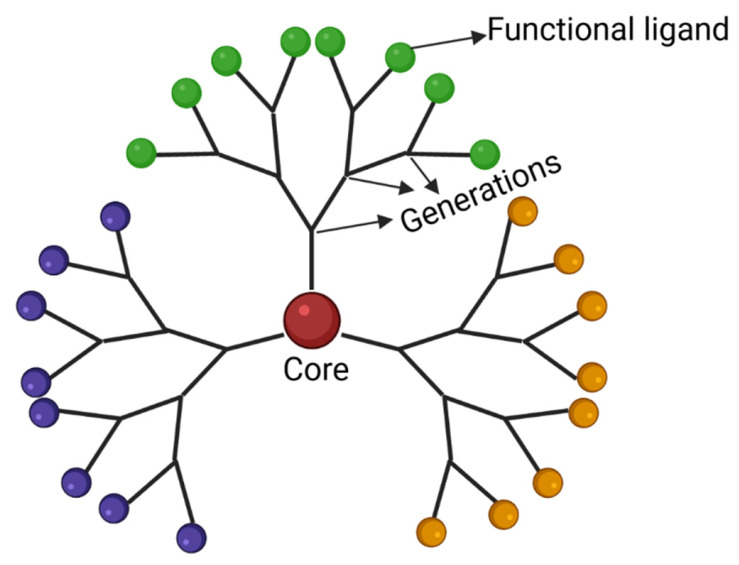
Dendrimers are NPs whose building blocks extend out in generations from a central core. The surfaces of these tree-like structures can be conjugated with a number of functional ligands that can target BBB and tumor receptors and carry chemotherapeutic drugs.

Dendrimers are multifunctional hyper-branched polymers with high molecular uniformity and monodispersity. Their structure consists of a core atom or group of atoms from which building blocks repeatedly extend, resembling a tree (Figure 2). The peripheral building blocks branch out in patterns known as generations [188]. Dendrimers offer improved control over size, shape, and physical properties compared to linear polymers [83]. Therapeutic agents can be either conjugated to dendrimers through covalent bonds or attached through electrostatic adsorption, and the surfaces of dendrimers at the endpoints of branches can be modified with a large number of functional groups and targeting ligands [189]. However, care must be taken to choose surface groups that confer low toxicity, and positively charged groups in particular are associated with cytotoxicity [188,189]. A wide variety of dendrimers are available for drug delivery, including polyamidoamine, polypropyleneimine, poly-L-lysine, and phosphorus dendrimers [145,149,189,190,191,192]. Therapeutic drugs are often conjugated via biodegradable amide or ester linkages using chemical spacers, with amide bonding providing greater stability and ester bonding providing improved control over drug release [193].

Gliomas can be targeted by dendrimers by modifying dendrimer surfaces with spacers or linkages to improve stability and bioavailability, conjugating ligands to target specific brain tissue, and conjugating therapeutic drugs to the dendrimer. Conjugated ligands of interest include Tf to target tumors, LDL receptor activators to cross the BBB, and tamoxifen to inhibit multidrug efflux transporters [194,195,196,197]. In addition, imaging agents can be conjugated for in vivo tracking and tumor diagnosis [196]. Dendrimers have been investigated to deliver an array of chemotherapeutic agents for brain tumors, including methotrexate, doxorubicin, and arsenic trioxide [144,149,198]. The drug-release kinetics can be improved by using an acid-sensitive linkage between the dendrimer and chemotherapeutic, ensuring pH-controlled release as acidity gradually increases in internalized endosomes [199]. Controlled release of doxorubicin has been accomplished by linking the drug to polyadmiodamine dendrimers using a cis-aconityl linkage, with PEGylation prolonging circulation time and further improving the drug-release kinetics [200,201]. As with other NPs, dendrimers can also be used for gene therapy and siRNA, with Bai et al. illustrating that a polyamidoamine dendrimer complexed with the interferon beta gene could induce apoptosis in mouse brain tumor cells [202].

### 4.4. Inorganic Metals

NPs can also be designed from metal compounds, allowing fine control over shape, size, and porosity [203]. Drugs and other ligands can be readily conjugated to their surfaces, and a variety of inorganic metals have been studied for drug delivery to the brain, including gold, silver, zinc oxide, iron oxide, and silica [204,205,206,207]. Metallic NPs can also behave as contrast imaging agents due to their high electron density [207,208,209]. However, compared to other NPs, metallic NPs often exert a significant cytotoxic effect on brain tissue, resulting in oxidative stress, autophagy, and a microglial inflammatory reaction, including upregulation of pro-inflammatory cytokines such as TNF-α, IL-1β, and IL-6 [210,211,212]. A study in rats found that exposure to zinc oxide NPs resulted in cognitive impairment, particularly in older mice, potentially from suppression of cAMP/CREB signaling [206]. Trickler et al. showed that the pro-inflammatory effect of silver NPs could be used to increase BBB permeability, with smaller silver NPs exerting the greatest inflammatory effect [204]. The cytotoxic effect could also be leveraged against brain cancer; however, care must be taken to avoid damage to healthy tissue.

The magnetic properties of metallic NPs are commonly exploited for novel treatments for brain cancer. The application of an external magnetic field can guide the NPs to precise locations, while alternating magnetic fields can be used to increase the internal temperature of magnetic NPs, producing a hyperthermic effect known as thermotherapy, which can destroy cancer cells [213,214,215]. Indeed, a human study found that thermotherapy could improve survival time in patients with recurrent glioblastoma, and no serious complications were observed [216]. In addition, thermotherapy can create mechanical stress on endothelial TJs and transiently increase permeability of the BBB to promote NP uptake [217].

**Figure 3:**
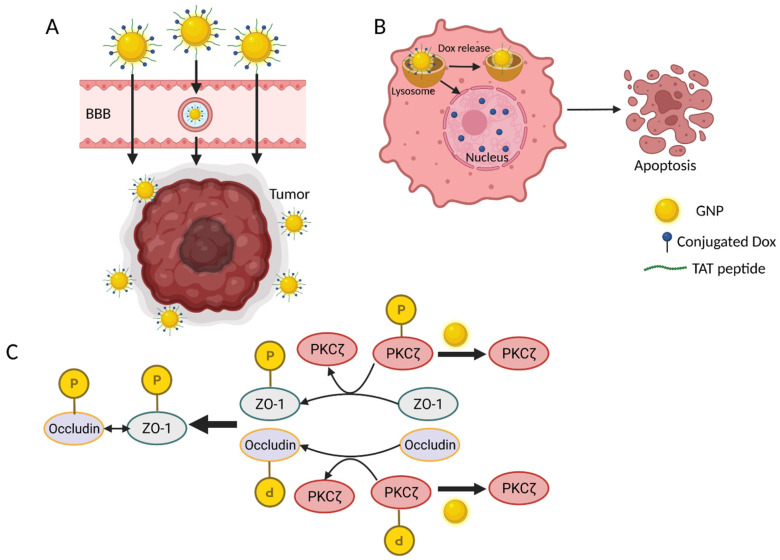
Transport of GNPs to tumor cells. (**A**) Gold NPs carrying the chemotherapeutic agent doxorubicin and the TAT peptide can cross the BBB through paracellular transport and AMT to reach tumor cells. The GNPs accumulate at the tumors via the enhanced permeability and retention effect. (**B**) After internalization by the tumor cell, the GNPs are transported into lysosomes, where doxorubicin is released from its hydrazone linkage by the acidic microenvironment. The doxorubicin enters the nucleus and damages DNA by acting as an intercalator, resulting in apoptosis of tumor cells. (**C**) GNPs improve paracellular transport across the BBB by decreasing the level of phosphorylated PKCζ, an enzyme required for the proper association between ZO-1 and occluding at the tight junctions of endothelial cells.

Gold nanoparticles (GNPs) have been extensively studied for targeting brain tumors as they are easily synthesized, stable, and can incorporate many surface molecules. Immunofluorescent staining of endothelial cells treated with GNPs has revealed discontinuous zonula occludens-1 adaptor proteins which normally stabilize endothelial TJs, while Western blotting has found decreased expression of occludins and phosphorylated PKCζ. Phosphorylation is critical for the active form of the PKCζ isozyme, which in turn phosphorylates zonula occludens-1 and occludins, resulting in their association at the TJ. By inhibiting activation of the PKCζ isozyme, GNPs may impair the structural integrity of TJs and improve BBB permeability to conjugated drugs [218]. The arginine-rich transactivator of transcription (TAT) peptide derived from HIV has been studied as a ligand that interacts favorably with the negatively charged endothelial membrane to further improve uptake of GNPs [219,220]. Chemotherapeutics can be loaded to the TAT-GNP conjugate, such as doxorubicin, which displays 3–14x greater cytotoxicity and improves survival time in mice compared to doxorubicin treatment alone (Figure 3) [152]. Doxorubicin can be conjugated via an acid-labile hydrazone linker, allowing release of the therapeutic agent within the acidic microenvironment of tumor cells while sparing healthy tissue. TAT-GNPs can also deliver gadolinium chelates as contrast agents for brain tumor imaging, with in vitro results demonstrating an 82-fold increase in gadolinium chelate concentration compared to the free chelate alone [152]. GNPs can also be used to deliver small interfering RNA molecules to cross the BBB and target oncoproteins to reduce tumor size in mice models [221].

### 4.5. Quantum Dots

Visualization of brain cells, biological processes, and pathological tissue can be achieved using NPs, particularly quantum dots (QDs), which are semiconductor nanocrystals capable of in vivo imaging [222,223,224]. Upon exposure to light, they fluoresce with tunable excitation/emission spectra depending on their size, shape, and composition [225]. Multicolor and multitarget imaging can be achieved using their broad excitation and narrow emission spectra [226]. QDs can visualize brain vasculature, neurons, and glial cells, and even individual receptors and ion channels [227]. Conjugation with ligands such as Tf and TAT improves their uptake across the BBB and can be used to target specific cells [227]. Carbon quantum dots have also been studied due to their biocompatibility and photoluminescent properties. They can be derived from precursor molecules such as glucose, where they have shown capability of crossing the BBB using glucose transporters without the need for conjugating targeting ligands [228]. 

QDs have been shown to infiltrate gliomas, offering the possibility of visualizing tumors in real-time [229]. Consequently, QDs may be used for preoperative tumor diagnosis, intraoperative visualization of tumor margins during surgical resection, and postoperative monitoring [230]. Given upregulation of the epidermal growth factor receptor in many tumors, antibodies to the receptor labeled with QDs were shown to selectively bind glioblastoma and olidodendroglioma tissue specimens overexpressing the growth factor receptor [226]. Visualization was achieved even at the single-cell level in live tissue and biopsies, and can clearly demarcate tumor boundaries [231,232]. NPs attached to DNA aptamers targeting the growth factor receptor can also cross the BBB and selectively accumulate in tumor cells to generate a strong fluorescent signal to visualize the tumor extent [230]. Other target receptors can be used for QD imaging, such as the TfR [233]. 

By conjugating chemotherapeutic agents, QDs can serve as both fluorescent probes and therapeutic drug carriers for otherwise impermeable drugs [227]. QDs generated from carboxymethylcellulose and conjugated with doxorubicin can function as photoluminescent probes for tumor imaging while selectively targeting glioblastoma cells with chemotherapy [234]. Carbon QDs conjugated with Tf and doxorubicin have also been shown at low concentrations in vitro to reduce viability of pediatric brain tumors by 14–45% across different cell lines, exhibiting greater cytotoxicity compared to free doxorubicin alone due to greater uptake and specificity [156]. Similarly, carbon QDs with paired α-carboxyl and amino groups can interact with the large neutral amino acid transporter 1 frequently upregulated in cancer cells. Results from a glioma mouse model illustrated selective uptake by glioma cells and near-infrared fluorescence and photoacoustic imaging of the tumors. Loading of the QDs with the chemotherapeutic topotecan resulted in a targeted killing of tumor cells while reducing toxicity to normal tissues compared to free topotecan [155].

### 4.6. Nanogels

Hydrogels are three-dimensional hydrophilic polymeric structures capable of holding large amounts of water without dissolving, resembling biological tissue [235,236]. A nanocomposite hydrogel can be formed by embedding NPs directly into a hydrogel network or gel matrix, allowing hydrogels to carry NPs. Nanogels can be designed as nanosized hydrogels, combining the unique advantages of hydrogels, including their fluid-like transport properties, low toxicity, serum stability, and uniformity, with the benefits of NPs, including small size, improved permeability, and intravenous administration [236,237]. These nanogels are bioadhesive, biocompatible, and biodegradable, feature high loading capacity, and are flexible and deformable [238,239]. Drugs can be released in a controlled fashion upon degradation of the nanogel [240]. Drug release can also be triggered by a specific stimuli, including pH level, ultrasound, or temperature [238].

Nanogels can cross the BBB and target tumor tissue in novel ways. Singh et al. used a diphtheria toxin receptor ligand for nanogel transcytosis across the BBB due to upregulation of the receptor on glioma blood vessels. The radioactive drug 5-[125I]Iodo-4″-thio-2″-deoxyuridine was released from the nanogel following degradation of the nanogel’s carbonate linkages in response to the glioblastoma’s acidic microenvironment [241]. Angiopep-2, a ligand that binds the LDL receptor, also improves permeability of nanogels across the BBB, allowing them to release doxorubicin to glioblastoma [158]. Nanogels can increase endocytosis of the chemotherapeutic drug methotrexate across the BBB by 10-fold compared to free methotrexate [160]. Nanogels can also carry miRNA that downregulates glioblastoma target genes and inhibits tumor growth, although this was studied using intratumoral injection, and its efficacy in crossing the BBB is unclear [242]. Newer methods include delivering gene therapy with CRISPR/Cas9 coupled with hydrogel NPs targeted to brain tumors to inhibit tumor growth [243].

The stimuli-responsiveness of nanogels in response to temperature has received considerable attention for neurological applications. These thermosensitive nanogels undergo a sol-to-gel transition, or a change from a liquid to gelatinous structure, at a target temperature, frequently body temperature [244]. At room temperature, the liquid nanogels can pass through a needle for injection, after which they assume a gelatinous form that allows for the controlled release of drugs and conforms to tissue shape [240]. Considerably more research has been performed using thermosensitive hydrogels compared to nanogels for treating brain cancers with therapeutics; however, similar principles apply. For example, the OncoGel is a hydrogel copolymer of poly(d,l-lactide-*co*-glycolide and PEG that can deliver the chemotherapeutic paclitaxel to glioma cells and prolong survival in rats [245]. In vitro research into thermosensitive nanogels carrying doxorubicin has illustrated their capacity to respond to higher tumor microenvironment temperatures to deliver therapeutics [246]. Thermosensitive hydrogels can also be used to improve localization of NPs to tumors, with Brachi et al. illustrating that gelation upon exposure to body temperature can improve uptake and retention of NPs by glioblastoma cells [247]. Similarly, Lin et al. designed thermosensitive hydrogels for delivery of chemotherapeutic drugs with bovine serum albumin NPs, showing increased survival in a mouse model of glioblastoma [161]. 

## 5. NPs in Neuro-Oncology

Non-invasive imaging and therapeutic treatment of tumors can be achieved with NP technology [248]. As an imaging tool, NPs can be used to aid in diagnosis, pre-operative planning, and monitoring of treatment response. The ability of NPs to specifically target tumor cells and carry fluorescent probes or contrast agents renders them valuable as imaging agents. NPs can carry gadolinium-based agents to improve contrast imaging of gliomas and reduce background noise on MRI [249]. Magnetic NPs can offer improved delineation of tumor margins, more intense contrast enhancement, and can accumulate in neoplasms for longer periods compared to gadolinium-based contrast agents, offering longer windows for acquisition [250]. Fe_3_O_4_ NPs are commonly used magnetic NPs that have found clinical use as MRI contrast agents to improve T2-weighted MR imaging, owing to their increased BBB uptake, tumor targeting, and shorter transverse relaxation time [249,251,252]. Similarly, manganese dioxide NPs that can respond to the tumor microenvironment have been explored to improve MR imaging of gliomas [253]. GNPs can be used to detect glioma cells under MRI and fluorescent microscopy, and can also highlight tumor microvasculature [254,255]. Liposomes can be used to transport lipid-binding fluorescent carbocyanine dyes that otherwise cannot cross the BBB for in vivo tumor imaging [176]. They can also target molecules upregulated in tumor angiogenesis, such as CD105, to depict the tumor neovasculature and monitor its progression [256]. Metallofullerenes, whose carbon cage confers a high degree of stability, can transport gadolinium-based contrast agents to improve tumor delineation and visualization on MRI and can be conjugated with IL-13 peptides to target glioma cells [257,258]. Fluorescent NPs, such as QDs, offer inherent optical properties that enable improved imaging of brain tumors [230]. NPs can also aid in photoacoustic imaging, which combines optical imaging from a pulsed laser with high-resolution ultrasound imaging [259]. Contrast agents for photoacoustic imaging can be derived from NPs, including a semiconducting polymeric NP that was shown to clearly visualize gliomas in mice [260]. 

Delivery of chemotherapeutic drugs to the brain is usually hindered by the impermeability of the BBB. Delivering chemotherapeutics from NPs is a novel strategy for treatment of brain tumors. Such therapeutic agents can offer reduced toxicity to patients by selectively accumulating in the target area of interest, thereby mitigating systemic side effects. In addition to therapeutic drugs, NPs can be loaded with aptamers and siRNA molecules as gene therapy for brain tumors [261]. Some clinical trials have been performed in humans with promising results, following earlier investigations in in vitro and in vivo models. Clinical investigations for brain tumors can also be conducted by adapting NPs used for treatment of tumors elsewhere in the body. For example, Caelyx is a marketed agent consisting of doxorubicin transported by a liposomal NP that has been used in treatment of breast and ovarian cancer. Consequently, it was investigated in rodent models of brain tumors, and successful results were followed by clinical studies establishing safety and efficacy, including prolonged survival in patients with recurrent high-grade gliomas [178,179]. Subsequently, researchers investigated modulations to further improve efficacy and conjugated glutathione to the liposomes to target the glutathione transporter on BBB endothelial cells, with in vitro and in vivo rodent studies illustrating inhibition of glioblastoma growth [262]. Finally, a Phase I/IIA clinical study of glutathione PEGylated liposomal doxorubicin (2B3-101) demonstrated antitumor activity in patients with brain metastases and high-grade gliomas without adverse neurotoxicity or cardiotoxicity, although long-term follow-up is unclear [263]. Human trials can also offer a more thorough exploration of the safety profile compared to animal models, with the aforementioned study finding gastrointestinal side effects as most common. Another Phase I trial of intravenous liposomal irinotecan found that dose-limiting toxicity included diarrhea [264]. Despite promising results, challenges remain in translating success from in vitro and animal models to human patients. For example, a Phase II study of PEGylated liposomal doxorubicin with temozolomide and radiotherapy for glioblastoma found the treatment to be feasible and safe, but did not meaningfully improve patient outcomes [265].

Imaging and treatment of brain tumors can be jointly carried out by NPs, an approach known as theranostics [208]. NPs used as imaging or contrast agents can be conjugated with drugs that target brain cancer, while others can be conjugated with both imaging probes and drugs. Such NPs are considered multifunctional, owing to their multiple functional units used to achieve discrete functions, including imaging, drug release, tumor targeting, and evasion of the reticuloendothelial system (Table 2) [266,267]. The fluorescent dye Cy5.5 can be attached to GNPs delivering doxorubicin to brain tumors, enabling fluorescent in vivo imaging to monitor drug delivery and treatment response [268]. NPs can also be combined for theranostic purposes. For example, hyaluronic acid nanogels that release doxorubicin to tumor cells can be crosslinked with fluorescent carbon dots for real-time tracking of drug delivery. The hyaluronic acid is used to target CD44 overexpressed on tumor cells; however, the researchers did not test the hybrid system in an in vivo brain tumor model [269]. Similarly, PEGylated liposomes containing QDs along with the chemotherapeutic agent docetaxel can be synthesized and coated with transferrin to improve permeability across the BBB, allowing treatment of brain tumors while monitoring liposomal distribution [270]. Such a system exploits the drug delivery and imaging properties of NPs, with their capacity to selectively target tumor cells via conjugation of unique functional groups. 

## 6. Limitations and Future Directions

Research in animal models of brain cancer has illustrated promising results for a diverse array of NPs. Nonetheless, there are several important limitations to consider prior to adoption for human use. Uptake of NPs or degradation outside the brain limits the availability of NPs that can cross the BBB and target brain tissue. After intravenous administration, NPs can be opsonized by serum albumin, complement components, immunoglobulins, and other plasma proteins, resulting in their recognition by cell-surface receptors of phagocytes, including macrophages. NPs are thereby sequestered in the spleen, lymph nodes, and liver by the reticuloendothelial system. Within macrophages, they are transported to lysosomes and degraded [281]. NPs that escape internalization and destruction may still be opsonized by macrophages, potentially obscuring targeting and functional ligands and reducing permeability across the BBB [281,282]. The physical properties of NPs, including size, charge, and composition, all affect circulation time in the bloodstream and should be carefully investigated. In addition to reduced tumor uptake, NPs bearing chemotherapeutic drugs can potentially harm the liver and spleen during sequestration [33]. Among other strategies, PEGylation is commonly performed to reduce NP destruction and improve serum half-life, and NPs can be designed to release their drugs only in response to specific stimuli [283].

Neurotoxicity is always a concern with administration of particles to the brain. NPs targeted to the brain can contribute to neuroinflammation by activating microglia, immune cells of the central nervous system that behave similarly to peripheral macrophages [284]. The activated microglia secrete reactive oxygen species and nitric oxide, resulting in excitotoxicity and neuronal damage [285]. Even NPs not specifically targeted to the brain have been shown to accumulate in neural tissue, where they may result in oxidative stress, DNA damage, and apoptosis, raising concern for long-term or routine exposure to NPs used in other biomedical and engineering applications [286,287]. In particular, metallic NPs have been shown to accumulate in the brain and cause inflammatory damage [288]. Silica NPs have been found to exert neurotoxic effects and injure dopaminergic neurons in the striatum [289]. Although this ordinarily constitutes a harmful response, the underlying mechanism can be exploited to reduce neuro-inflammation by targeting activated microglia with NPs [290]. In vivo animal studies have generally validated the safety of NPs designed specifically for treatment or imaging of brain tumors. Still, consideration must be afforded to the NP dose, composition, and physical and chemical properties [291]. Some studies have illustrated an increased toxicological effect with cationic NPs compared to anionic NPs [38]. Furthermore, by increasing the permeability of the BBB, NPs may also result in uptake of undesired toxic substances [79].

NPs can be targeted to unique stimuli of tumor microenvironments, including hypoxia, protein biomarkers, and a low pH, and can respond to external signals, such as magnetic fields to produce magnetic hyperthermia or ultrasound for photoacoustic therapy [292]. The cancer microenvironment is a complex milieu that often features simultaneous changes in genetic expression of multiple biomarkers, and NPs can be engineered to respond only in the presence of multiple environmental inputs together [293,294]. These NPs contain molecular logic gates that can be modeled with Boolean logic for imaging of tumors and therapeutic drug release [295]. Side effects are minimized and specificity is improved by incorporating multiple stimuli. Such logic-gated systems can, for example, be represented in the spatial arrangement of NPs and differential responses to target tissue [295]. Functional moieties that respond to external stimuli by changing their conformation can be targeted to cancer-specific biomarkers [293]. QDs have been explored for molecular logic-gating by modulating their degree of fluorescence in response to surrounding molecules [296]. Similarly, Badeu et al. showed that adding stimuli-responsive synthetic cross-linkers to hydrogels can allow fine control over hydrogel degradation and drug release in the presence of multiple stimuli, including enzymes, light, and reductants [294]. Aptamers have also been used for assembly of “DNA nanorobots” that change their structure in response to environmental cues [296]. Ma et al. showed that a DNA nanorobot acid can target breast cancer tumor cells to promote degradation of membrane proteins [297]. Ongoing research into molecular logic gating may improve the efficacy of NPs for brain tumors.

NPs represent a promising means of improving drug uptake across the BBB, allowing for improved imaging and treatment of malignant brain tumors. The poor outcomes still seen with malignant brain tumors are a testament to the challenges associated with development of effective brain delivery systems. Ongoing investigation is necessary to develop brain delivery systems that can be translated to human trials. The optimal physical and chemical properties of NPs, including size, charge, and functional groups, should be interrogated and optimized in the laboratory setting. In vitro and in vivo models can help determine if a NP is effective at treating brain tumors with low toxicity to healthy tissue. Intravenous administration of NPs should be compared to administration of the free drug alone to determine the differential effect of the NP formulation. Given that NPs often accumulate in the reticuloendothelial system, analysis of the biodistribution of a NP under investigation is warranted to ensure selective accumulation at the tumor site. Furthermore, adjunct mechanisms to improve tumor targeting should be explored, such as FUS for improving BBB permeability, or magnetic fields for targeting magnetic NPs to tumors. Other delivery mechanisms for NPs can be pursued, including convection-enhanced delivery and intranasal delivery. After establishing safety and efficacy in animal models, human clinical trials should be performed to determine pharmacokinetics, tolerable therapeutic windows, and finally effectiveness at treating brain tumors. Moreover, NPs may be combined with conventional anti-tumor methods, such as surgery, radiation, and chemotherapeutics to maximize efficacy. A diverse array of permutations can be achieved with NPs, and rigorous investigation is necessary to determine those agents that should achieve use in humans.

## 7. Conclusions

Malignant brain tumors confer a poor prognosis, and chemotherapeutic treatments are limited by the impermeability of the BBB. NPs represent a versatile platform for novel treatment paradigms for malignant brain tumors. Their small size, low toxicity, multifunctionality, and modifiability make them valuable agents for targeting brain tumors. NPs can cross the BBB through paracellular transport, carrier-mediated transport, and transcytosis, and can be targeted to tumor cells with ligands overexpressed on endothelial and tumor cells. Several categories of NPs have been studied for BBB transport and tumor treatment, and consideration should be afforded to their unique physical and chemical properties, including size, charge, and associated ligands. NPs can also be used as imaging agents to produce higher-resolution scans of tumors, demarcate tumor boundaries and margins, and monitor drug delivery and therapeutic response. Theranostic NPs are capable of integrating disparate functions, including drug delivery with in vivo imaging. Results from animal studies demonstrate the promise of NPs for improving outcomes in patients suffering from brain tumors, and continued investigation is necessary to translate these findings into routine practice.

## Figures and Tables

**Table 1:** Summary of NPs and select chemotherapeutic ligands investigated for treatment of brain tumors. In addition, NPs are capable of delivering RNA and DNA molecules as gene therapy and can play roles as imaging agents for tumors.

Nanoparticle Category	Advantages	Chemotherapeutics	References
Polymeric	Stability, biodegradability, biocompatibility, ease of manufacturing, hydrophobic and hydrophilic drug transport, non-immunogenic, low toxicity	3-bis(2-chloroethyl)-1-nitrosourea	[130,131]
		Doxorubicin	[132,133]
		Methotrexate	[134]
		Temozolomide	[135]
		Gemcitabine	[136]
		Paclitaxel	[137]
Liposome	Hydrophobic and hydrophilic drug transport, biocompatible, low toxicity	Doxorubicin	[120]
		Methotrexate	[138]
		Cisplatin	[139]
		Irinotecan	[140,141]
		Topotecan	[142]
		Paclitaxel	[143]
Dendrimer	High molecular uniformity, monodispersity, kinetic stability, abundant free functional groups, low toxicity	Methotrexate	[144]
		Doxorubicin	[145,146]
		Temozolomide	[147]
		Docetaxel	[148]
		Arsenic trioxide	[149]
Metallic	Contrast imaging agents, surface is readily modifiable, inflammatory cascade increases BBB permeability, hyperthermic effect increases BBB permeability and damage tumors	Doxorubicin	[150,151,152]
		Cisplatin	[153]
		Paclitaxel	[154]
Quantum dots	Photoluminescent, photostability, tunable emission/excitation spectra, visualization of individual molecules, readily monitor drug delivery, low toxicity	Topotecan	[155]
		Doxorubicin	[156]
		Temozolomide	[157]
Nanogels	Serum stability, uniformity, fluid-like transport properties, bioadehsive, biocompatible, biodegradable, deformable, stimulus-responsive release, low toxicity	Doxorubicin	[158]
		Cisplatin	[159]
		Methotrexate	[160]
		Paclitaxel	[161]

**Table 2:** Studies on theranostic NPs, investigated for brain cancer, which combine a therapeutic anti-cancer effect with tumor imaging.

NP Category	Size (nm)	Functional Components	Model	Results	Refs
Liposome + QD	182	Docetaxel (chemo), QD (imaging), transferrin (targeting)	Rats	Sustained drug release >72 h	[270]
Carbon Dots	6–8	Highly crystalline carbon nanodot (photoacoustic imaging and photothermal therapy)	Mice (U87 glioma cells)	NPs accumulate in tumor cells and image-guided near-infrared-activated photothermal therapy can damage tumor tissue.	[271]
Magnetic NP	12	Epirubicin (chemo), Fe_3_O_4_ core (contrast imaging)	Rat (C6 glioma cells)	FUS can improve uptake across the BBB, magnetic targeting improves tumor targeting, and MRI can monitor magnetic NP distribution.	[272]
Silica NP	__	Doxorubicin (chemo), Cu_2 − *x*_ Se NP (photoacoustic imaging)	Mice (U87 glioma cells)	FUS can improve uptake across the BBB for tumor-specific targeting and the NPs show contrast enhancement on imaging.	[273]
GNP	56	Doxorubicin (chemo), Cy5.5 (probe), RRGD peptide (targeting)	Mice (C6 glioma cells)	Effective uptake by glioma cells with co-localization and fluorescent detection of Cy5.5	[268]
Gold + iron oxide-loaded micelle	100	Iron oxide (MRI contrast agent), GNP (radiosensitizer)	Mice (U251 GBM cells)	Effective contrast agent for MRI and can show tumor borders of glioblastoma, radiosensitization increases tumor damage from radiation therapy	[274]
Iron oxide NP	43	Iron oxide (MRI contrast agent), IL1- receptor antagonist (anti-edema agent)	Rats (C6 glioma cells)	IL-1 receptor antagonist reduces peritumoral edema and improves survival, enhanced MRI imaging of tumor	[275]
Iron oxide NP	37	Iron oxide (MRI contrast agent), Small interfering RNA (gene therapy), temozolomide (chemo)	Mice (T98G GBM cells)	Gene therapy can reduce glioblastoma resistance to temozolomide, therapeutic response can be monitored on MRI	[276]
Iron oxide NP	184	Iron oxide (MRI contrast agent), doxorubicin (chemo)	C6 glioma cells	MRI showed NP accumulation in tumor cells	[277]
Gadolinium-based NP	120	Chlorin e6 (photosensitizer), gadolinium (MRI contrast agent)	Mice (C6 glioma cells)	Photodynamic therapy targeted tumor cells, NPs showed contrast enhancement on MRI	[278]
Polymeric NP	40–70	Doxorubicin (chemo), gadolinium or Hoechst 33342 (imaging agents)	Mice (breast cancer metastasis line)	MRI and fluorescence microscopy confirmed delivery of imaging agents across the BBB, doxorubicin induced apoptosis in metastatic cells	[279]
Polymeric NP	10–200	Iron oxide (MRI contrast agent), Photofrin (photosensitizer), F3 peptide (targeting)	Rat (9 L glioma cells)	Photodynamic therapy increased survival time, MRI detected NPs in tumor cells	[280]

## Abbreviations

AMT	Adsorptive-mediated transcytosis
BBB	Blood-brain barrier
BBTB	Blood-brain tumor barrier
CNS	Central nervous system
FUS	Focused ultrasound
GNP	Gold nanoparticle
LDL	Low-density lipoprotein
MRI	Magnetic Resonance Imaging
NP	Nanoparticle
PBCA	Poly(butyl cyanoacrylate)
PEG	Polyethylene glycol
PLA	Poly(lactic acid)
QD	Quantum dots
RES	Reticuloendothelial system
RMT	Receptor-mediated transport
SLN	Solid lipid nanoparticle
TAT	Transactivator of transcription
Tf	Transferrin receptor
TfR	Transferring receptor
TJ	Tight junction
